# Endogenous bioelectric currents promote differentiation of the mammalian lens

**DOI:** 10.1002/jcp.26074

**Published:** 2017-08-30

**Authors:** Lin Cao, Jie Liu, Jin Pu, J. Martin Collinson, John V. Forrester, Colin D. McCaig

**Affiliations:** ^1^ Institute of Medical Sciences, School of Medicine, Medical Sciences and Nutrition University of Aberdeen Aberdeen UK; ^2^ Department of Ophthalmology First Hospital Affiliated to the Chinese PLA General Hospital Beijing P.R. China

**Keywords:** ATP1B1, differentiation, extracellular electrical signaling, lens epithelial cells, lens fiber

## Abstract

The functional roles of bioelectrical signals (ES) created by the flow of specific ions at the mammalian lens equator are poorly understood. We detected that mature, denucleated lens fibers expressed high levels of the α1 and β1 subunits of Na^+^/K^+^‐ATPase (ATP1A1 and ATP1B1 of the sodium pump) and had a hyperpolarized membrane potential difference (V_mem_). In contrast, differentiating, nucleated lens fiber cells had little ATP1A1 and ATP1B1 and a depolarized V_mem_. Mimicking the natural equatorial ES with an applied electrical field (EF) induced a striking reorientation of lens epithelial cells to lie perpendicular to the direction of the EF. An EF also promoted the expression of β‐crystallin, aquaporin‐0 (AQP0) and the Beaded Filament Structural Protein 2 (BFSP2) in lens epithelial cells (LECs), all of which are hallmarks of differentiation. In addition, applied EF activated the AKT and CDC2 and inhibition of AKT reduced the activation of CDC2. Our results indicate that the endogenous bioelectrical signal at the lens equator promotes differentiation of LECs into denucleated lens fiber cells via depolarization of V_mem._ Development of methods and devices of EF application or amplification in vivo may supply a novel treatment for lens diseases and even promote regeneration of a complete new lens following cataract surgery.

## 1 INTRODUCTION

**Figure 1:**
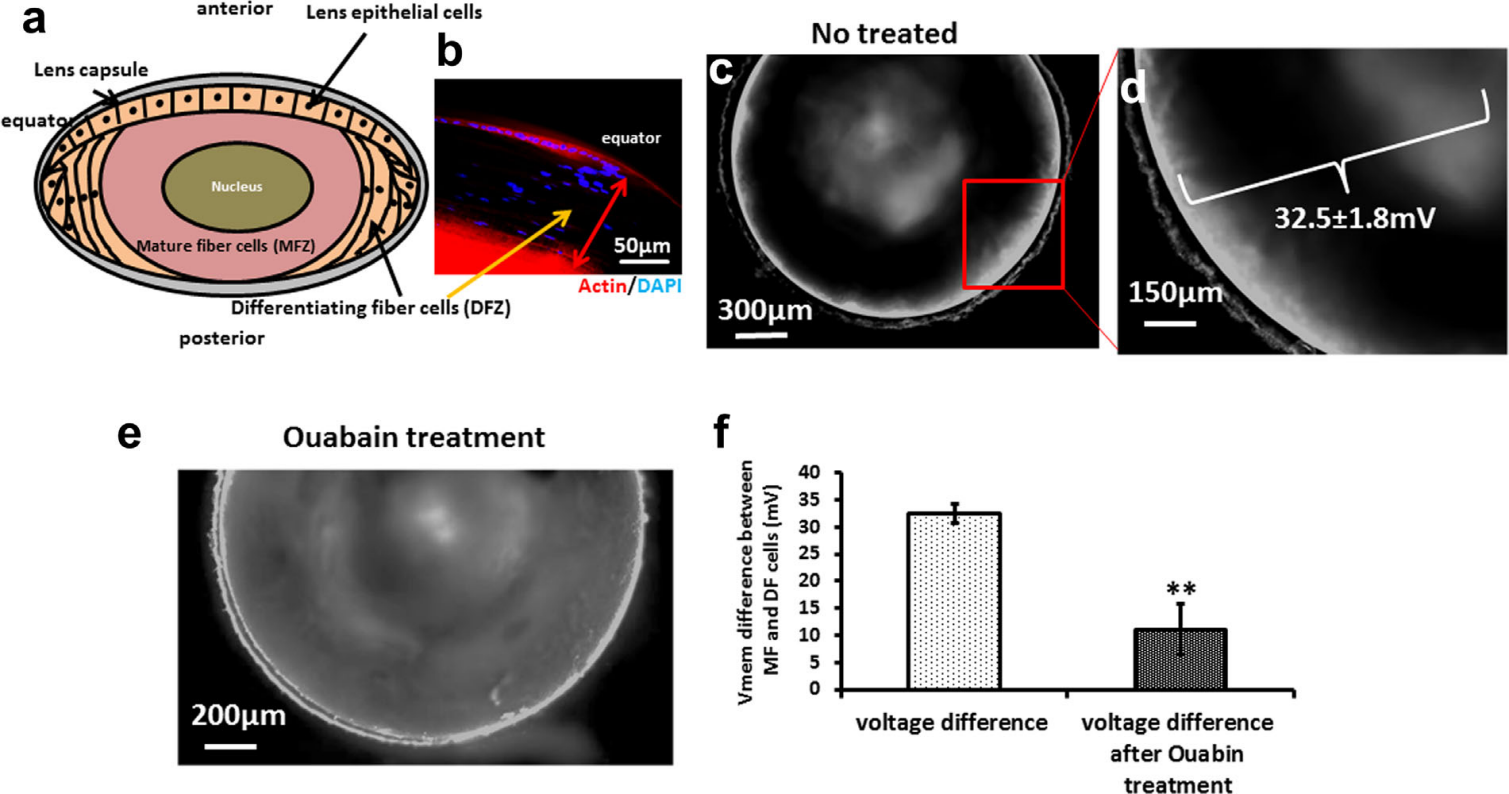
Lens DFZ cells have depolarized V_mem_ and MFZ cells are hyperpolarized. (a) Diagram of lens structure showing the differentiating fiber zone (DFZ) and mature fiber zone (MFZ). (b) The lens equator section was stained by DAPI and phalloidin‐TRITC (red) and shows that actin was expressed in LECs and in MFZ cells (red). The cells in the intervening DFZ (with nuclei stained blue with DAPI) expressed very much less actin. The width of DFZ is ∼120 μm (red arrow headed line). (c,d) Mouse lens treated with 5 μM DiBAC4(3) for 20 min and imaged from above . The DFZ area at the periphery of the lens shows cells with fluorescent staining. This indicates a depolarized V_mem:_ Further in from the periphery, MFZ cells did not fluoresce, indicating hyperpolarized V_mem;_ and depolarization of V_mem_ in the center of lens. (e) Lens treated for 1 hr with 30 μM ouabain before staining with DiBAC4(3). The hyperpolarized V_mem_ in the MFZ is reduced markedly as indicated by the more uniform fluorescent staining throughout both DFZ and MFZ. (f) We measured the intensity of the fluorescence gradient across the DFZ and MFZ stained with DiBAC4(3) and calculated the potential difference as 32.5 ± 1.8 mV in untreated lenses and as 11 ± 4.7 mV in lenses treated with ouabain. There are two lens in each experiment and measurements were repeated three times

The ocular lens is transparent and comprises two cell types: a monolayer of lens epithelial cells (LECs) which forms a cap at the front and the highly elongated lens fiber cells (LFCs), which differentiate from LECs at the lens equator. Proliferation of LECs is restricted to a “germinative zone” at the equator (Sellitto, Li, & White, 2004; White, Gao, Li, Sellitto, & Srinivas, 2007; Rajagopal et al., 2008) and epithelial cells move through the germinative zone and into the “transitional zone” below the equator, where they withdraw from the cell cycle and differentiate into secondary fiber cells (Piatigorsky, 1981) (Figure 1a). This involves synthesis of lens fiber‐specific proteins (e.g., α‐ and β‐crystallin) and morphologic changes such as a highly oriented cell elongation (Piatigorsky, 1981). At subsequent stages of differentiation, fiber cells destroy their cell nuclei and other organelles, forming an organelle‐free zone (OFZ) in the central region of the lens that minimizes light scatter (Bassnett, 1995; Wormstone & Wride, 2011). Finally, a cascade of regulated proteolytic events enables the lens fiber cells to pack tightly together and the lens core to exclude water (Korlimbinis, Berry, Thibault, Schey, & Truscott, 2009; Lampi et al., 1998; Lampi, Shih, Ueda, Shearer, & David, 2002; Liu, Xu, Gu, Nicholson, & Jiang, 2011; Ueda, Duncan, & David, 2002), while fiber cells within the same growth shell fuse (Shestopalov & Bassnett, 2000, 2003). This epithelial to fiber cell differentiation process is ongoing throughout life, is promoted by the Wnt‐Fz/PCP (Wnt‐Frizzled/Planar Cell Polarity) signalling pathway (Chen, Stump, Lovicu, & McAvoy, 2006; Chen et al., 2009) and also by a gradient of fibroblast growth factor (FGF) (Lovicu & McAvoy, 2005; Robinson, 2006; Zhao et al., 2008) and is unique to lens. Although lens induction has been studied for over 100 years, much remains unknown about the many extracellular signaling pathways and gene regulatory networks orchestrating these processes.

The transmembrane potential difference (V_mem_) is the voltage drop across a cell membrane (typically −10 mV to −90 mV), and it contributes to functions such as migration, proliferation, and differentiation (Sundelacruz, Levin, & Kaplan, 2009). The V_mem_ is established by ionic gradients which arise by active and passive ion transport through membrane‐embedded ion channels and transporters, such as the Na^+^/K^+^‐ATPase, the so called sodium pump. Although maintenance of ionic homeostasis is a critical feature of cell metabolism and viability, surprising specificity has been uncovered in the relationship between changes in V_mem_ and the regulation of differentiation and cell death (Bortner & Cidlowski, 2004; Franco, Bortner, & Cidlowski, 2006; Sundelacruz et al., 2009).

Extracellular electrical gradients also regulate cell migration, proliferation, differentiation, and regeneration (McCaig, Rajnicek, Song, & Zhao, 2005). Lens generates extracellular electric currents (50–100 µA cm^−2^) that flow outward only at the equator and which re‐enter the lens at anterior and posterior poles. A K^+^ gradient based on spatial variations in Na^+^/K^+^‐ATPase activity and K^+^ channels in lens epithelium underpins the generation of the resulting electrical signal (ES) that is focused at the equator (Eperon, Rodriguez‐Aller, Balaskas, Gurny, & Guex‐Crosier, 2013). Here, we show that the extracellular ES together with the V_mem_ at lens equator play multiple physiological roles that regulate lens development, differentiation, and regeneration (Sundelacruz et al., 2009) and which collectively may be capable of building a lens.

## 2 RESULTS

### 2.1 The V_mem_ is depolarized in differentiating lens fiber cells

The resting membrane potential in non‐excitable cells (V_mem_) regulates important cellular properties such as proliferation, migration and differentiation and in development it varies spatially and temporally (Blackiston, McLaughlin, & Levin, 2009; Sundelacruz et al., 2009; Yang and Brackenbury, 2013). The negatively charged slow membrane potential reporter dye bis‐(1,3‐dibutylbarbituric acid)‐trimethine oxonol (DiBAC3(4)) becomes embedded in the lipid bilayer of the membrane and accumulates in the cytosol upon membrane depolarization (Kolosova, Lebedev, Fursova, Moroskova, & Gusarevich, 2003). Using mouse lens we showed that there was a clear spatial gradation from depolarization to hyperpolarization in passing from lens epithelial cells through to the differentiating fiber zone (DFZ, ∼120 μm wide immediately underneath the epithelial layer), and on further to mature fiber zone cells (fully differentiated fibers, MFZ) (Figure 1a–d). Slow potentiometric dyes (cationic or anionic) show fluorescence dynamics of ∼1% per mV (Kolosova et al., 2003). Therefore, since the fluorescence intensity in the DFZ zone was 32.5% greater than in MFZ, the difference in V_mem_ between the DFZ cells and the MFZ cells would be about 32.5 ± 1.8 mV (Figure 1d). Furthermore, the voltage gradient was reduced to about one third of this, to 11 ± 4.7 mV, by exposure to the specific Na^+^/K^+^‐ATPase inhibitor ouabain (30 μM) for 1 hr, with the fluorescent density of MFZ cells reduced to 11 ± 4.7% (Figure 1e,f). Clearly, the electrical gradient depends on the activity of the Na^+^/K^+^‐pump.

### 2.2 The expression levels of Na^+^/K^+^‐ATPase underpin depolarization of V_mem_ in differentiating lens fibers

**Figure 2:**
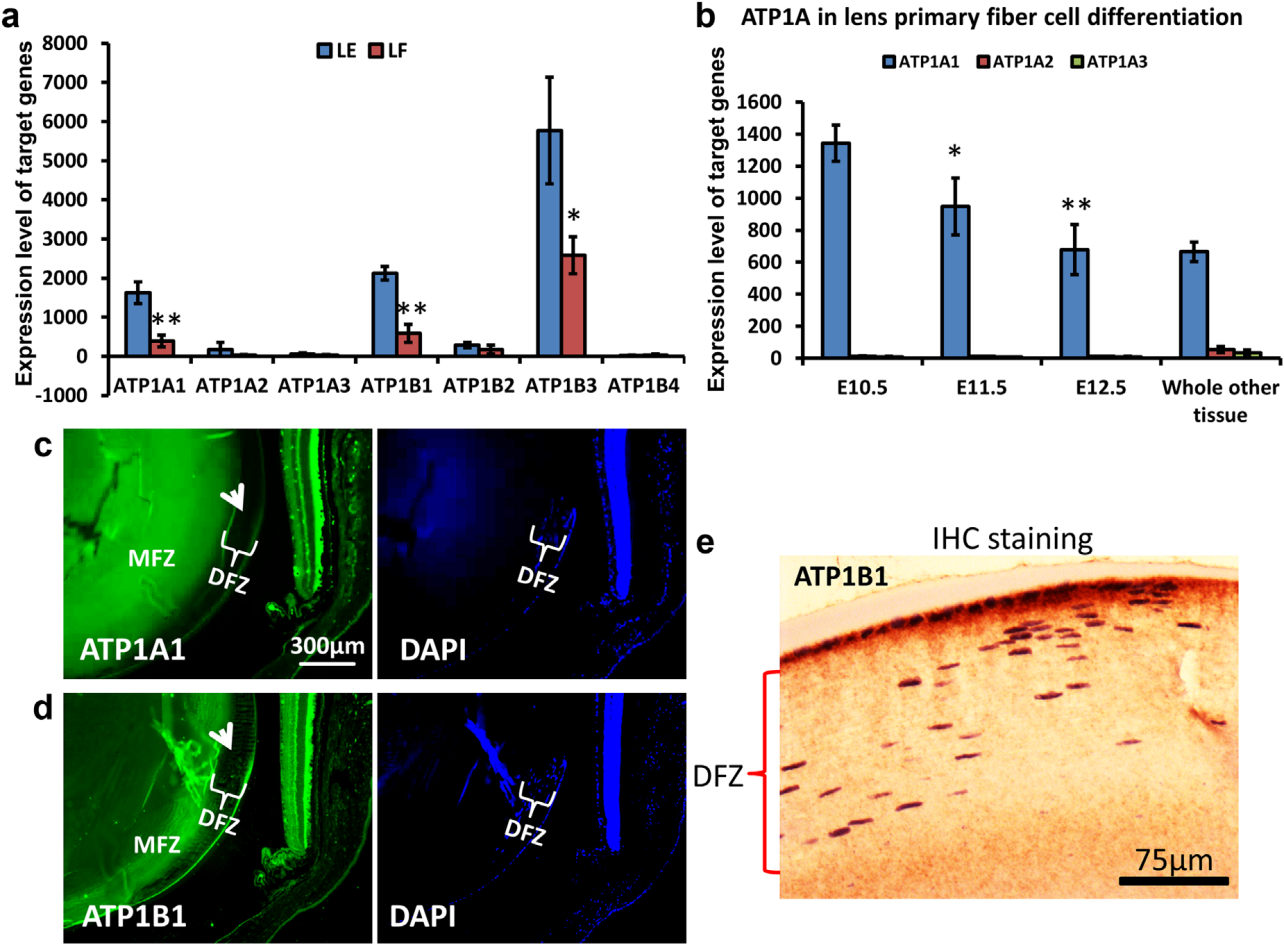
The expression and location of Na^+^/K^+^‐ATPase in lens epithelial and lens fiber cells. (a) From analysis of microarray data (GDS, the expression of the α1‐, β1‐ and β3‐subunit of the Na^+^/K^+^‐ATPase (ATP1A1, ATP1B1, and ATP1B3) all were reduced significantly in lens fibers of mice, compared to lens epithelial cells. (b) In GDS4452, analysis of lens from ICR strains of mouse embryos at three key developmental stages in the transition from E10.5, lens placode invagination to E12.5, lens primary fiber cell differentiation and matched whole embryo body was made. The results showed that ATP1A1 and ATP1B3 were reduced significantly in lens fiber cells at E12.5. *p < 0.05, **p < 0.01. (c,d) The expression of ATP1A1 and ATP1B1 by immunofluorescence (green, white arrow heads) staining showed reduced expression in DFZ cells in mice lens with nuclei. ATP1A1 and B1 were over‐expressed in MFZ and cells were without nuclei. Blue staining is DAPI for nuclei. (e) Immunohistochemistry staining showed significant expression of ATP1B1 in lens epithelial cells and MFZ cells, but low levels of expression in DFZ cell in mouse lens

To determine the origin of the ES in lens, we analyzed the expression of Na^+^/K^+^‐ATPase subunits from microarray data on GEO (www.ncbi.nlm.nih.gov/geo). The data showed down‐regulation of ATP1A1 and ATP1B1 and ATP1B3 (β3‐subunit of Na^+^/K^+^‐ATPase) in differentiating lens fibers cells compared to lens epithelial cells (Figure 2a), with the reduced expression of ATP1A1 being dependent on the developmental stage of embryonic mice (Figure 2b). Because Na^+^/K^+^‐ATPase activity contributed to the V_mem_ (McCaig et al., 2005), we next stained longitudinal sections of mouse lens to determine the expression level of the Na^+^/K^+^ ATPase pump subunits ATP1A1 (α1‐subunit of Na^+^/K^+^‐ATPase) and ATP1B1 (β1‐subunit of Na^+^/K^+^‐ATPase). We found that ATP1A1 and B1 were expressed most prominently in mature (differentiated) lens fiber cells and in lens epithelial cells, but remarkably, hardly at all in differentiating zone lens fibers (Figure 2c–e). Our data indicate that the location of Na^+^/K^+^‐ATPase is cell type dependent, is regulated spatially and generates a membrane potential that may instruct lens cell differentiation.

### 2.3 Applying an EF to mimic the electrical signal at the equator induced reorientation of lens epithelial cells

**Figure 3:**
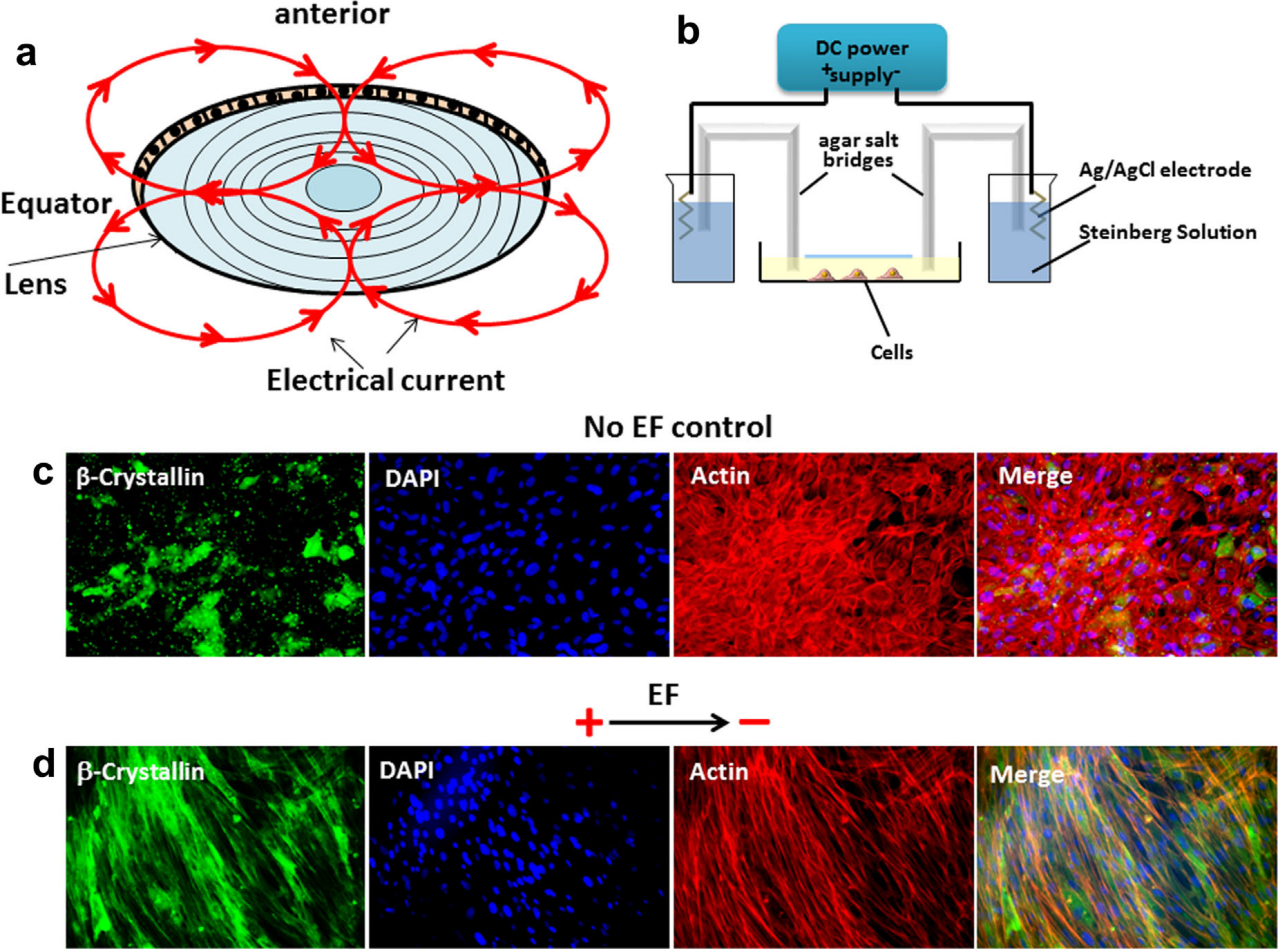
An applied EF induced lens epithelial cells to reorient roughly perpendicular to the electric field. (a) Diagram of lens electrical current flow shown by the red circles with arrowheads. The electrical current is flowing outward at the equator and inwards at anterior and posterior poles. (b) Diagram of the chamber used to apply the EF to the cells. (c) No electric field, the cells showed multiple polygonal shapes without elongation in any one axis (images are taken after 24 hr in culture). (d) In an applied EF of 100 mV/mm for 24 hr, lens epithelial cells re‐aligned with a markedly elongated axis perpendicular to the EF and with increased expression of β‐crystallin

At the equator of developing and mature lens, polarized ion transport creates an extracellular electrical signal (ES). Electrical current flows outward at the lens equator only and re‐enters via the anterior and posterior poles (Figure 3a) (Eperon et al., 2013; McCaig et al., 2005). As lens epithelial cells move slowly toward the equator multiple stages of differentiation occur, the early ones include cell elongation and re‐orientation. Applying an EF to mimic the electrical current flow at the equator remarkably induces lens epithelial cell elongation and re‐orientation to lie perpendicular to the EF (Figure 3c,d). This suggests that the electrical current at the equator may act as a reorientation signal for lens epithelial cells as they begin the differentiation processes that will transform them into lens fiber cells.

### 2.4 An electrical signal promotes differentiation of human lens epithelial cells

**Figure 4:**
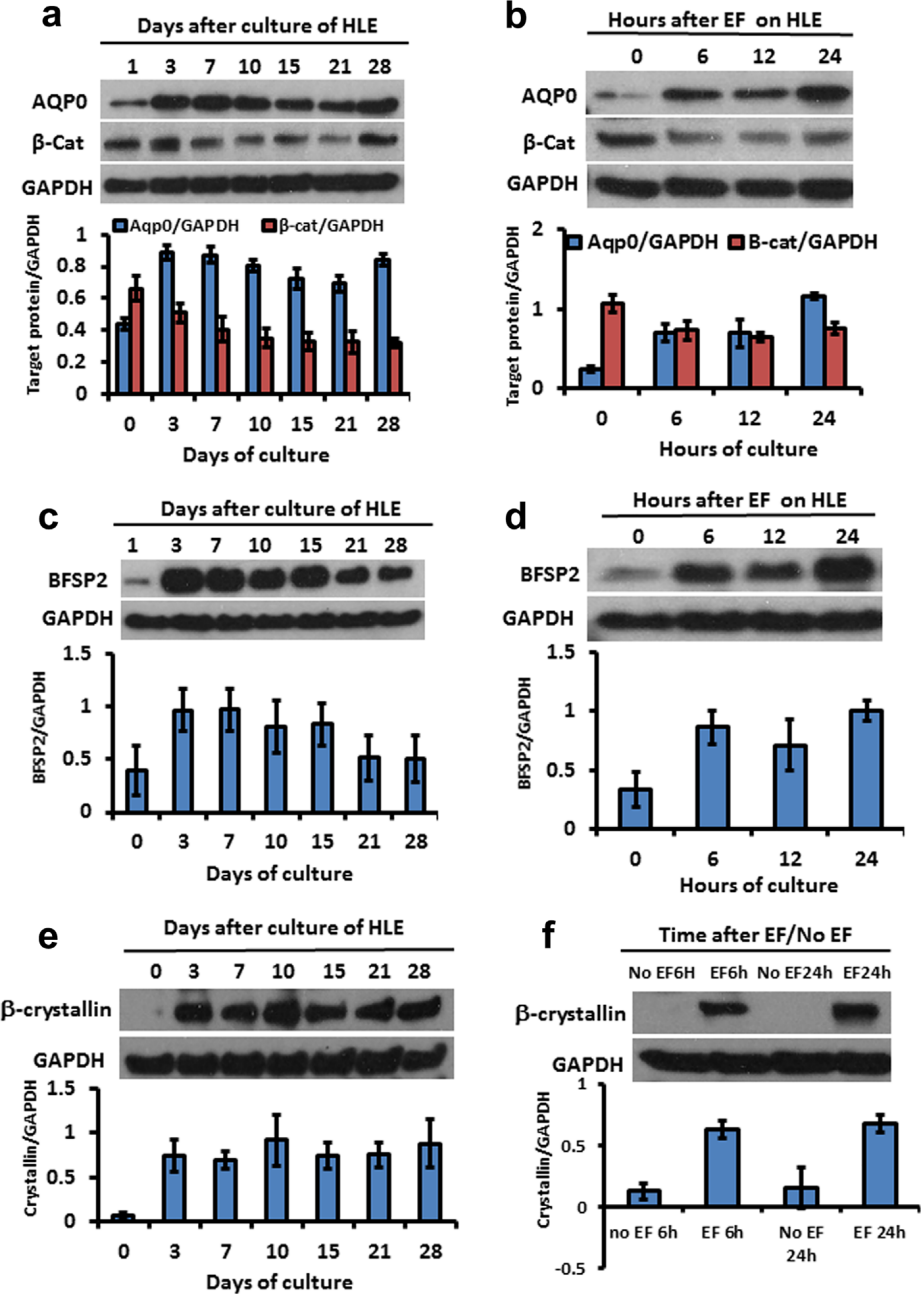
Mimicking the lens electrical signal promoted differentiation in human lens epithelial cells (HLECs). (a–d). HLECs were harvested at different culture times for western blotting. The expression of AQP0, BFSP2 and crystalline increased after 3 days and was maintained in general throughout 28 days indicating sustained differentiation. β‐catenin as a marker of epithelial cells was reduced after 7 days culture. This also heralds the shift from epithelial cells to lens fiber cells. When the HLECs were exposed to an applied EF in vitro, the expression of AQP0 and BFSP2 as markers of differentiation increased significantly within only 6 hr and the expression of β‐catenin also was inhibited by EF treatment within 6 h. (e,f) β‐crystallin expression which took around 3 days to increase in control cultures not exposed to an EF, showed a marked increase within 6 hr in cultures exposed to an applied EF. The diagrams under each electrophoresis image are normalized optical density of each band as a ratio relative to GAPDH

During differentiation, lens epithelial cells express a range of specific proteins at specific times. AQP0 is the most abundant protein in the plasma membrane of lens fiber cells where its functions include acting as a water pore, fiber cell–fiber cell adhesion and control of fiber cell structure and organization (Bhat, 2001). In contrast, the intermediate filament protein BFSP2 (Beaded Filament Structural Protein 2) is expressed only after fiber cell differentiation (Patterson, 1988; Vaghefi, Liu, & Donaldson, 2013). The lens epithelial protein, β‐catenin, promotes lens cell proliferation and initiates fiber cell differentiation, and polarization but is dispensable once fiber elongation and differentiation of the fiber cell in the lens cortex has taken place. Meanwhile, β‐crystallin is expressed in lens development and forms the major cytoplasmic protein of the lens. In cultured human lens epithelial cells strong/peak expression of all four proteins occurred after 3 days (Figure 4a,c,e) while the early expression of β‐catenin began to decline by ten days (Figure 4a), mirroring to an extent the in vivo developmental biochemical changes. Application of an EF (100 mV/mm) to cultured LECs altered the kinetics of protein expression markedly by accelerating the appearance of lens differentiation proteins. Elevated expression of AQP0, and BFS2 was detectable as early as 6 hr and reached maximum levels by 24 hr of culture, rather than 3 days without the EF (Figure 4b,d) and similar changes were observed with β‐crystallin (Figure 4f). By contrast β‐catenin expression levels began to decline after 6 hr of culture in an EF (Figure 4b). These data support strongly the concept of a major role for a physiological EF in promoting and regulating lens cell differentiation.

### 2.5 Electrical signals activate AKT/CDC2

**Figure 5:**
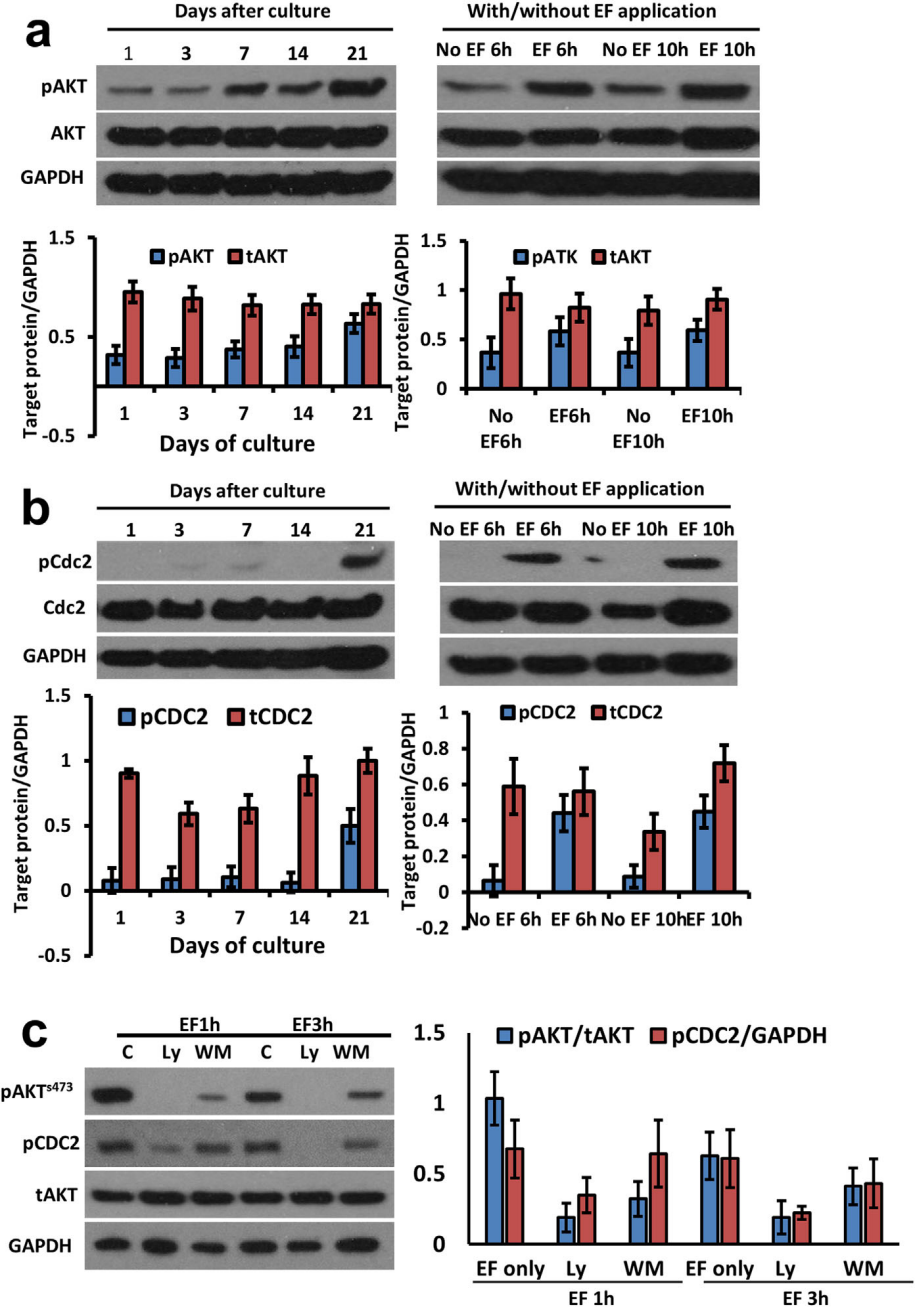
An EF activated AKT and CDC2 in lens epithelial cells. (a) AKT was activated in BLECs after 7 days in control cultures, but this took only 6 hr when exposed to an EF of 100 mV/mm. (b) Similarly CDC2 was activated in 21 days in BLEC control cultures, but this took only 6 hr when exposed to an applied EF. (c) inhibition of AKT by ly294002 and wortmannin blocked the activation of CDC2. The diagrams under each electrophoresis image are normalized optical density of each band relative to GAPDH

**Figure 6:**
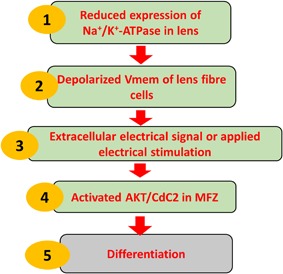
Model for electrically directed lens differentiation. Reduced expression of Na^+^/K^+^‐ATPase leads to a depolarized V_mem_ and an extracellular electrical signal which becomes focused at the equator. An applied EF which mimics the electrical signal activated AKT/CDC2 and mediated lens epithelial cell differentiation

CDC2‐dependent phosphorylation is required to initiate nuclear membrane disassembly during mitosis, a precursor to the removal of the nucleus which occurs during lens fiber cell differentiation (Bhat, 2001). We found that phospho‐AKT (pAKT) and phospho‐CDC2 (pCDC2) were elevated over several weeks in control cultures (no EF), but that remarkably an applied EF stimulated elevated levels of both pAKT and pCDC2 much more rapidly, within as little as 6 hr (Figure 5a,b). In addition, inhibition of AKT blocked the activation of CDC2 in lens epithelial cells (Figure 5c). This indicates that EF‐induced lens fiber differentiation leading to denucleation may be mediated by the AKT/CDC2 signaling network (Figure 6).

## 3 DISCUSSION

Throughout adult life, lens epithelial cells transdifferentiate into lens fiber cells through migration, proliferation in organized parallel arrays, elongation, and denucleation (McCaig et al., 2005). As fiber differentiation is a major event in lens morphogenesis, much effort has been focused on determining how this is regulated. Because proliferation and differentiation occur throughout life, the lens is an attractive developmental model, but how a tissue or organ develops its characteristic size and three‐dimensional cellular architecture is poorly understood. Here, we identified that the ES present at the lens equator has a central signaling role in orchestrating many aspects of lens differentiation of fiber cells.

### 3.1 Na^+^/K^+^‐ATPase levels regulate cell depolarization and the electrical current at lens equator

The transmembrane potential difference (V_mem_) is the voltage gradient across the plasma membrane that is established by the balance of intracellular and extracellular ionic concentrations (Sundelacruz et al., 2009). Increasing evidence has pointed toward not only a correlation, but a functional relationship between V_mem_, extracellular electrical signals and cell functions such as proliferation and differentiation. Here, we detected that mature, denucleated lens fibers expressed high levels of the α1 and β1 subunits of Na^+^/K^+^‐ATPase (ATP1A1 and ATP1B1) and had hyperpolarized cell membrane potential differences (V_mem_). In contrast, differentiating, nucleated lens fiber cells had little ATP1A1 and ATP1B1 and a depolarized V_mem_. Our data suggest that a depolarized V_mem_ may contribute to differentiation of lens fiber cells. The V_mem_ plays a role in maintenance of the differentiated phenotype in human mesenchymal stem cells pre‐differentiated toward osteoblasts or adipocytes (Sundelacruz, Levin, & Kaplan, 2013). Depolarization‐induced differentiation was found in PC12 cells to be mediated by phospholipase D2 (Banno et al., 2008). Understanding the basis of biophysical regulation will highlight novel ways to direct cell functions and harness biophysical signalling for regenerative medicine and tissue engineering (Sundelacruz et al., 2009).

At the equator of developing and mature lens polarized ion transport, driven by cells with varying V_mem_, creates an extracellular ES. Electrical current flows outward at the lens equator only and re‐enters via the anterior and posterior poles (Figure 3a) (Eperon et al., 2013; McCaig et al., 2005). This creates a standing extracellular electrical gradient in the lens. We have visualized this electrical gradient using a potentiometric dye. The negatively charged slow membrane potential reporter dye bis‐(1,3‐dibutylbarbituric acid)‐trimethine oxonol (DiBAC3(4)) becomes embedded in the lipid bilayer of the membrane and accumulates in the cytosol upon membrane depolarization (Kolosova et al., 2003). Our data showed in mouse lens that there was a clear electrical gradient from depolarization to hyperpolarization in passing from lens epithelial cells through to the differentiating fiber zone and on further to mature fiber zone cells. It also indicates that the extracellular ES that exists between DFZ and MFZ is the consequence of both transmembrane and extracellular ion gradients. The strength of this signal between the DFZ cells and the MFZ cells is about 32.5 ± 1.8 mV (43.3 ± 2.4 mV/mm).

In addition, the lens equator is a highly specialized region which regulates the sequential switches between lens epithelial cell migration, proliferation, and differentiation. Electric current flows outward only at the lens equator and inward at the anterior and posterior poles (Bhat, 2001; McCaig et al., 2005; Robinson & Patterson, 1982; Wang et al., 2005). Vaghefi et al. (2013) established a computer model to predict the steady state properties of the lens and showed that current efflux from lens fibers is highly concentrated at the equator, causing net current flow to be outward (Patterson, 1988). Using vibrating probe technology, outward electrical currents of 50–100 μA/cm^2^ have been measured at the equator (Robinson & Patterson, 1982; Wind, Walsh, & Patterson, 1988) and the DFZ region has a resistance of 3,000 Ω^•^Cm which doubles to around 6,000 Ω^•^Cm in the MFZ (Mathias, Rae, & Baldo, 1997). By Ohm's law, the strength of the EF at the equator therefore is around 30 mV/mm in DFZ and 60 mV/mm in MFZ, consistent with the 32 mV which we detected using a fluorescent technique. The high resistance in the MFZ and DFZ is critically important for EF generation by maintained ionic gradients and is analogous to the tight junction regulated transepithelial resistance which maintains the voltage gradient across an epithelium (the transepithelial potential difference).

### 3.2 Mimicking the lens electrical signals induced lens fiber differentiation

Importantly, mimicking the natural electrical signals of the lens equator by applying a physiological EF (100 mV/mm) to LECs in culture induced many of the cellular events that occur around the lens equator (McCaig et al., 2005; Wang, Zhao, Forrester, & McCaig, 2003;). Our data and previous research have identified that an EF directed LEC migration, promoted cell elongation and cell reorientation (Wang, Zhao, Forrester, & MCCaig, 2000), regulated cell cycle progression to mitosis and oriented epithelial cell division along the EF vector (McCaig et al., 2005). Furthermore, an applied EF increased the expression of β‐crystallin, AQP0 and BFSP2, all markers of lens fiber differentiation. Clearly the EF not only has a functional role in the directed migration and reorientation of lens epithelial cells, but also regulates their differentiation into lens fiber cells. This indicates that the endogenous voltage gradient (electrical signal) at the equator creates a micro environment which acts to signal lens fiber differentiation. It points also to the importance of restoring and maintaining an endogenous electrical signal at the equator following lens removal in order to promote lens regeneration (Lois et al., 2010).

### 3.3 Electrically‐induced differentiation is mediated by activation of AKT and CDC2

Depolarization by treating with 100 mM KCl for 5 min resulted in the undulating phosphorylation of GSK‐3[beta] at Ser‐9 in SH‐SY5Y human neuroblastoma cells, in H19 −7/IGF‐IR rat embryonic hippocampal cells, and in PC12 rat pheochromocytoma cells (Lee et al., 2005). CDC2 (Cell Division Cycle 2) or CDK1 (Cyclin‐Dependent Kinase 1,) −dependent phosphorylation is required to initiate nuclear membrane disassembly during mitosis, a precursor of nuclear removal during fiber cell differentiation (Bhat, 2001). Park et al. propose that a *PTEN*–PI3 K/AKT–p21–CDK1 pathway regulates the cell cycle and cell death (Park et al., 2008). The presence of phosphorylated PKB (AKT) on the centrosome at the time of GVBD (germinal vesicle breakdown) suggests an important role for an initial CDK1 (CDC2) activation (Kalous et al., 2006). Collectively, these observations suggest a potential link between membrane depolarization and activation of AKT and CDC2 in differentiation of lens fiber cells. Therefore, we assessed the activation of AKT and CDC2 in an applied EF and found that both phospho‐AKT (pAKT) and phospho‐CDC2 (pCDC2 or pCDK1) were elevated within as little as 6 hr and maintained over at least 10 hr, but that without an EF, these signaling elements took 7 and 21 days respectively to be activated. These data indicate that the activation of AKT and CDC2 may mediate the depolarization‐induced differentiation by extracellular electrical signal at the lens equator.

### 3.4 Potential clinical implication

The mammalian lens regenerates provided the lens capsule is left behind after lentectomy and crucially provided that closing the lens capsule restores the normal electrical signals to the lens/capsular bag (Lois et al., 2010). In vertebrates, especially in adult mammals, lens can be regenerated in rabbits (Gwon, Gruber, & Mundwiler, 1990), in cats (Gwon, Gruber, & Mantras, 1993), in rats (Lois et al., 2010) and in mice (Call, Grogg, Del Rio‐Tsonis, & Tsonis, 2004) provided the lens capsule is left behind after lentectomy. Lois et al. (2010) reported that such a newly regenerated lens was optically clear and biochemical analysis showed a pattern of expression of lens development proteins at 8 weeks after lentectomy. Here we found that an EF promoted the differentiation of lens epithelial cells indicating that regulating the endogenous EF may promote lens regeneration and reduce the time for lens regeneration. EF application therefore might be considered as a feasible option after lentectomy and may even regenerate a physiological lens capable of accomodation and superior therefore to intraocular lens implants.

In addition, cataract is the most common cause of blindness (Ibaraki, 1997; Thylefors, Negrel, Pararajasegaram, & Dadzie, 1995) and failure to form the OFZ results in a cataractous lens. There is an increase in membrane permeability of lens cells with age that leads to an increase in internal Na^+^ and Ca^2+^ ions due to a reduced activity of Na^+^/K^+^ ATPase in cortical cataract and diabetes. This leads to overhydration, protein loss, and an increased lenticular Na^+^ and Ca^2+^ and decreased K^+^ content in cataract (Sanderson, Marcantonio, & Duncan, 2000). We found that an applied EF effectively increased the expression of crystallin, AQP0 and BFSP2 which are all proteins of lens fibers and that the EF also promoted OFZ formation. Perhaps an applied EF represents a novel way to prevent and treat cataract.

Collectively our discoveries of electrical regulation of lens development and of full lens differentiation, indicate a pivotal role for lens electrical signals perhaps even as a master regulator in building a lens both developmentally and during regeneration. There is growing interest in endogenous bioelectric signals/and how these may be exploited to control stem cell behavior and to develop better therapeutics (Sundelacruz, Levin, & Kaplan, 2015). Our data and that of others make it probable that humans will regenerate a lens, provided the challenge of restoring its bioelectric signals following surgical lens removal is met by carefully closing and resealing the capsular bag.

## 4 METHODS AND MATERIALS

### 4.1 Lens epithelial cell isolation and culture

The human LEC line (B‐3) was from ATCC (USA) and was cultured in Eagle's minimum essential medium EMEM (Sigma, UK) with 20% fetal calf serum (Sigma‐Aldrich, Irvine, UK). In addition, primary cultured bovine lens epithelial cells (BLECs) were isolated and cultured from bovine eyes as described previously (Bhuyan & Bhuyan, 1994). In brief, bovine lenses were obtained from eyeballs shortly after the animals were killed humanely. A small cut was made in the posterior capsule of the lens, the free edge was grasped with forceps, and the capsule with attached epithelium was placed in a 60 mm tissue culture dish (Corning, NY). The epithelium was cut into two or three fragments, and each fragment was placed in a separate dish. Three milliliters of DMEM (Sigma, UK) containing 20% inactivated fetal calf serum (Sigma, UK) and penicillin–streptomycin solution (0.01%) were added and cultures were maintained at 37°C in a water‐saturated air atmosphere containing 5% CO_2_, with the medium changed twice weekly.

### 4.2 Membrane potential dye staining

The voltage‐sensitive dye DiBAC3(4) (Thermo fisher Scientific, Perth, UK) was used to determine the membrane potential in lens tissue and in cultured cells. Each sample was washed in buffered Hank's salt solution then incubated for 20 min in 5 μM DiBAC3(4) at 37°C. After being rinsed three times with fresh buffer, stained samples were mounted on an inverted Zeiss microscope (Axiovert 135 TV). Dual wavelength images of excitation at 440 and 530 nm were acquired using a cooled CCD camera (Photometrics, Model CE200A, Tucson, AZ). Auto‐fluorescence signals were negligible at both excitation wavelengths compared with DiBAC3(4) fluorescence. All microscopy experiments were performed at room temperature.

### 4.3 Immunofluorescent staining and imaging

Cells were fixed in 4% paraformaldehyde for 20 min, followed by permeabilization (5 min) and blocking (30 min). The cells were stained for 2 hr with antibodies to α‐ and β‐subunit of Na^+^/K^+^‐ATPase (EMD Millipore, Watford, UK), α and β‐crystallin (BD Biosciences, Oxford, UK), respectively and then were incubated with secondary antibodies (Invitrogen) and phalloidin‐TRITC (Sigma–Aldrich) for 1 hr. Images were obtained with the Zeiss Axio Observer Z1 inverted fluorescence microscope (Carl Zeiss, Germany).

### 4.4 Immunohistochemistry staining

Mouse eyes were fixed with 2% paraformaldehyde (Agar Scientific Ltd. Cambridge, UK) for 2 hr. After paraffin embedding the eyeballs were cut into 5 μm thick sections and mounted on charged glass slides. Slides were de‐paraffinized and subjected to citrate‐based antigen retrieval. Paraffin sections were retreated with DAKO high pH antigen retrieval system (DAKO, Carpinteria, CA) using a domestic 600 kW microwave oven. Nonspecific antibody binding was blocked by incubating sections in 4% BSA, followed by 10% nonimmune goat serum (Zymed Corp., San Francisco, CA). Primary antibody was applied at a 1:200 to 400 dilutions overnight at room temperature. Sections then were incubated with secondary antibody for 30 min. The localization of target proteins was demonstrated with pre‐diluted streptavidin‐horseradish peroxidase (Zymed, UK) and 0.05% 3, 3‐diaminobenzidine in TBS, with H_2_O_2_ as the substrate. All sections were counterstained lightly with hematoxylin.

### 4.5 Applied electrical stimulation

Direct current (DC) electric fields (EFs) used to mimic the endogenous ES were applied to primary cultured LEC cells in 2 × 2 cm electrotaxis chambers as previously (Figure 3b) (Cao et al., 2014). In brief, a DC EF of 100 mV/mm was applied and measured directly (34410A digital multimeter, Agilent Technologies, Harrow, UK). Samples were exposed to an applied EF for 6–24 hr, then fixed for IF staining or prepared as cell pellets for protein assays.

### 4.6 Western blotting

Western blotting was performed as described (Wind et al., 1988). Primary antibodies used include anti‐AQP0 (Abcam, Cambridge, UK), BFSP2 (EMD Millipore), β‐Catenin (BD Biosciences), α‐ and β‐Crystallin (Life technologies, Paisley, UK), and GAPDH (Santa Cruz Biotechnology, Dallas, TX). The immunoblots were detected by Clarity Western ECL Substrate (Bio‐Rad, Watford, UK). For applied EF stimulated experiments, LECs were cultured in a specially designed chamber described previously (Wang et al., 2000). Cells were left unstimulated overnight to adhere to the dish and then an EF of 100 mV/mm was applied for variable times with/without treatment by 50 μM Ly294002 (Cell Signaling Technology, Danvers, MA) and 0.5 μM wortmannin (Sigma‐Aldrich). Cell lysates were collected for WB experiments.

### 4.7 Microarray data analysis

The microarray data sources were from the Gene Expression Omnibus (GEO) (Chen et al., 2008). Two data sets (series accession number of GDS1327 and GDS4452) which had been normalized when we obtained them were not subjected to any additional normalization. In GDS1327, cells were micro‐dissected from lens of post mortem donors. Then the human lens epithelial cells and lens cortical fiber cells (*n* = 6) were collected routinely and analyzed with Affymetrix Human Genome U133A array (Andley, Rhim, Chylack, & Fleming, 1994). In GDS4452, analysis of lens from ICR strains of mouse embryos at three key developmental stages in the transition from E10.5 lens placode invagination to E12.5 lens primary fiber cell differentiation and matched whole embryo body was made and then total RNA was extracted for probing the entire genome on Affymetrix Mouse Genome 430 2.0 Array ENREF (Barrett & Edgar, 2006). The identity of genes across microarray data sets was established using public annotations, primarily based on Unigene.

### 4.8 Statistical analysis

A minimum of three replicates was performed and analyzed for each experiment presented. Data are presented as the mean ± s.e.m. Student's *t*‐test was used to assess the significance and differences were considered as statistically significant with a *p*‐value <0.05.

## CONFLICTS OF INTEREST

The authors declare no competing financial interests.
